# Prospective single-arm study of gastric surgery in experimental animals assisted by Kangduo surgical robot

**DOI:** 10.1097/JS9.0000000000005035

**Published:** 2026-03-16

**Authors:** Songtao Yu, Yuliuming Wang, Tianyi Ma, Tianyu Qiao, Hao Zhang, Yunxiao Liu, Chunlin Wang, Wenyang Li, Chenkai Zhang, Haonan Qi, Yuxuan Li, Zijing Guan, Bowen Chi, Ruoyu Liu, Yinghu Jin, Ziming Yuan, Guiyu Wang

**Affiliations:** Department of Colorectal Cancer Surgery, The Second Affiliated Hospital of Harbin Medical University, Harbin, Heilongjiang, China

**Keywords:** animal experiment, Kangduo Surgical Robot 2000, partial gastrectomy

## Abstract

**Objective:**

To investigate the feasibility, efficacy, and safety of gastric surgery in experimental animals assisted by the domestic Kangduo Surgical Robot 2000 (SR2000).

**Methods:**

Eight porcine models were prospectively enrolled between August and December 2023 at Harbin SAGEBOT Intelligent Medical Animal Experiment Center. Preoperative baseline characteristics, intraoperative physiological parameters, surgical procedure, and postoperative assessments and investigations were recorded to compare the feasibility, efficacy, and safety evaluation indexes of experimental animals in the perioperative period. The primary endpoint was surgical success rate. The secondary endpoints were operative workflow parameters, intraoperative blood loss, postoperative laboratory changes, perioperative adverse events, and surgeon workload assessed using the NASA-TLX instrument.

**Results:**

In eight experimental pig models, the SR2000 successfully assisted in the completion of partial gastrectomy. All surgeries were completed according to plan, with no intermediate open or intraoperative complications, and the perioperative survival rate of animals was 100%. The mean operation time of partial gastrectomy was 76.75 min (range: 50–105 min), and the positioning time was 6–17 min. Intraoperative bleeding was ≤5 ml in all the animals, and some animals showed elevated white blood cell counts and neutrophil counts (Neu) on the first day of the postoperative period. The liver and kidney function indexes (alanine aminotransferase, blood urea nitrogen, creatinine) were not significantly abnormal before and after surgery. The anastomosis healed well without complications such as infection and anastomotic fistula.

**Conclusion:**

The feasibility, efficacy, and safety of the SR2000 were verified in animal experiments, and ergonomically optimized design have the potential for clinical dissemination.

## Background

The advent of robotic-assisted surgery systems has significantly enhanced minimally invasive surgical techniques, offering substantial advantages such as superior precision, enhanced stability, and improved ergonomics, particularly in complex gastrointestinal procedures^[^1–3^]^. While the da Vinci Surgical System currently dominates this field globally, with over 10 000 units deployed, its high procurement and operational costs have limited its adoption in China to approximately 500 units^[^4^]^. This economic barrier has driven the development of cost-effective domestic alternatives, aiming to broaden access to advanced robotic surgery within China and beyond^[^5^]^. Among these, the Kangduo Surgical Robot system, developed by Suzhou Kangduo Robot Co., Ltd., represents a significant stride in indigenous innovation.

HIGHLIGHTS

- Deployment-oriented animal experiment to de-risk Kangduo Surgical Robot 2000 clinical rollout in gastric surgery.
- Workflow and human-factors (NASA-TLX) assessment to guide training and credentialing.

**Figure 1.**
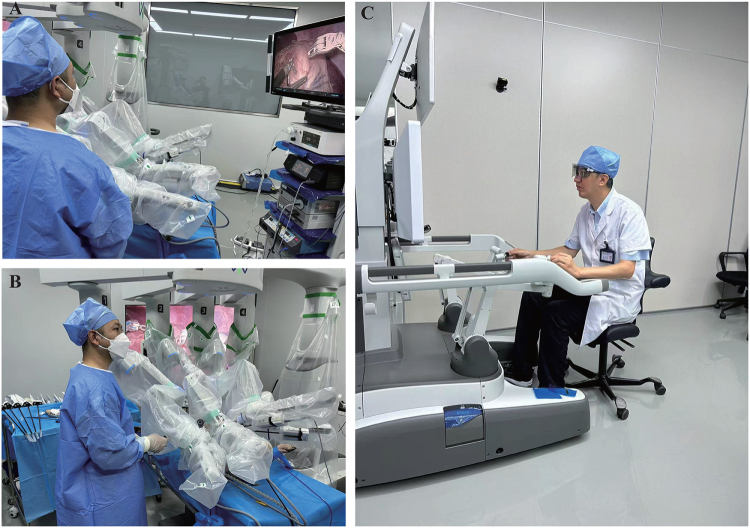
Kangduo SR2000 endoscopic surgical robotic system. (A) Patient cart docked to the porcine model with draped robotic arms; monitor shows the laparoscopic field. (B) Close-up view of multidegree-of-freedom instrument arms docked through trocars (C) A surgeon performs the operation using the Kangduo SR2000 endoscopic surgical robotic system.

The first-generation Kangduo robot (SR1000) has achieved notable milestones. It was the first domestically developed laparoscopic surgical robot approved under China’s “Innovative Medical Device Special Review Procedure.” Prospective randomized controlled trials across multiple Chinese hospitals demonstrated a 100% technical success rate in key procedures like partial nephrectomy and bowel resection^[^6,7^]^. Comparative clinical evaluations confirmed SR1000’s safety and efficacy as comparable to the da Vinci system, with its open console design offering potential ergonomic benefits for surgeons. By 2024, SR1000 had been successfully utilized in over 700 clinical cases spanning urology, general surgery (including gastrointestinal surgery), thoracic surgery, and gynecology, maintaining a stable perioperative complication profile and garnering positive recognition^[^8–10^]^. Building upon this foundation and seeking to address a wider range of clinical needs, Suzhou Kangduo Robot developed the next-generation Kangduo SR2000. This enhanced system features an open surgeon console, multi-degree-of-freedom robotic arms, and a 3D imaging system (Fig. 1), but critically upgrades to incorporate four robotic arms (compared to SR1000’s typical three) and support dual-console operation, significantly improving intraoperative flexibility, instrument triangulation, surgical exposure, and potential for collaborative surgery or training^[^11^]^. Although the predecessor SR1000 system has been successfully implemented in clinical practice, these substantial design changes necessitate dedicated preclinical testing to verify docking workflow, ergonomics, and instrument kinematics specific to the new platform. Therefore, an independent animal study is required before initiation of human trials, despite the clinical success of the earlier SR1000 system.

Recognizing the unique challenges of gastric surgery, such as intricate anatomy and complex reconstruction, rigorous preclinical evaluation of the enhanced SR2000 platform in this specific context is essential prior to widespread clinical adoption^[^12–14^]^. Therefore, this study aimed to conduct a systematic preclinical assessment of the domestically developed SR2000 endoscopic surgical robot system. Using a porcine model for partial gastrectomy procedures, we prospectively evaluated the system’s safety, effectiveness, and feasibility in gastrointestinal surgery. This analysis provides crucial preclinical evidence to inform and support the subsequent clinical translation of the SR2000 platform, while simultaneously contributing to national goals of fostering independent innovation and industrialization within China’s high-end medical device sector.

## Materials and methods

This study was completed in August–December 2023 at the Harbin SAGEBOT Intelligent Medical Animal Experiment Center, and the study was approved by The Second Affiliated Hospital of Harbin Medical University and the animal committee of this experiment center (Approval No.:SYDW2023-097). All procedures adhered to the ARRIVE 2.0 guidelines^[^15^]^. In addition, in line with the Transparency in the Reporting of Artificial Intelligence (TITAN) guideline, we explicitly disclose whether any AI systems were used in the conduct of the study or in manuscript preparation^[^16^]^.

### Study design and prespecified endpoints

This was a prospective single-arm study conducted in a porcine model. The primary endpoint was surgical success rate. Secondary endpoints included operative workflow parameters (positioning time, robotic arm operation time, and total surgery time), intraoperative blood loss, postoperative laboratory changes, perioperative adverse events, and surgeon workload assessed using the NASA-TLX instrument.

Before the *in vivo* procedures, both the primary surgeon and assistant completed standardized training on the SR2000 platform.

1. Laboratory animals

Eight Bama miniature pigs were selected and provided by Harbin SAGEBOT Intelligent Medical Animal Experiment Center. Animals were housed individually in stainless-steel pens under controlled environmental conditions (temperature 22 ± 2°C, humidity 40–60%, 12-h light/dark cycle). Environmental enrichment (rubber toys, bedding straw) was provided. Animals had free access to water and standard pig chow except during preoperative fasting.

2. Methods of operation

Preoperative preparation: 12 h fasting before surgery, no water for 6 h, measurement of animal weight, body temperature, and other routine check items.

Animal anesthesia: half an hour before the operation, using pentobarbital sodium and other anesthetics, in accordance with the routine use of the dose of general anesthesia to the animal, open the venous access, successful anesthesia, supine position, tracheal intubation, ventilator-assisted respiration, pentobarbital sodium to maintain anesthesia, real-time dynamic monitoring of the depth of anesthesia and vital signs indicators.

### Surgical operations

**Figure 2.**
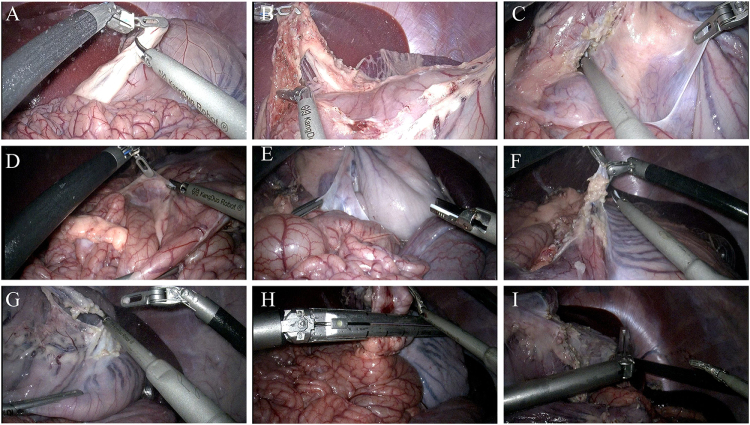
Robot-assisted laparoscopic partial gastrectomy. (A–C) Division of the gastrocolic ligament along the greater curvature with entry into the lesser sac (omental bursa) to achieve exposure, followed by distal dissection toward the antrum to approximately 4–6 cm proximal to the pylorus. (D–F) Skeletonization and division of the right gastroepiploic vessels along the greater curvature, then division of the short gastric vessels, with meticulous preservation of the splenic hilum and pancreatic tail. (G–I) Partial gastrectomy performed with a linear cutting stapler while preserving the cardia.

1. The test animals were fixed on the operating table in supine position, and the surgical area was prepared, routinely sterilized and toweled. An opening was made at the lower edge of the umbilicus at about 2 cm as an auxiliary hole, and 10 mm Trocar was inserted under direct vision, CO_2_ was filled to establish a pneumoperitoneum, and the air pressure was maintained at 14 mmHg. A laparoscope was inserted to locate the surgical site within the abdominal cavity, and to determine the position of the perforation for the lens-holding arm, which was at a distance of about 12–18 cm from the surgical site, and an opening was made for the hole in the left and right sides at 8–10 cm, and 10 cm was inserted respectively. With the hole position of the mirror arm as the center of the left and right 8–18 cm, 10 mm Trocar was inserted as the operation hole and connected to the robotic arm of the laparoscopic surgical system to enter the abdominal cavity, respectively.
2. Partial gastrectomy operation: After establishing pneumoperitoneum, the gastrocolic ligament was incised along the greater curvature beginning at the mid-body of the stomach to enter the omental bursa. Dissection was carried proximally toward the gastric fundus and distally to a point 4–6 cm proximal to the pylorus. The right gastroepiploic vessels and the short gastric vessels were sequentially divided, with meticulous preservation of the splenic hilum and pancreatic tail. A 36–40 Fr bougie was introduced orally along the lesser curvature to serve as a calibration guide. At 4–6 cm from the pylorus, vertical gastric resection along the bougie was initiated using a linear cutting stapler. Sequential stapler firings were performed proximally toward the esophagogastric junction, ensuring preservation of the cardia to minimize the risk of postoperative stenosis or gastroesophageal reflux. The staple line was inspected for bleeding, and hemostasis was achieved using absorbable sutures or titanium clips when necessary. Continuous seromuscular suturing was performed along the staple line to reinforce it and reduce the risk of postoperative leakage. In accordance with oncologic principles, the resected gastric specimen was enclosed in a specimen retrieval bag and removed through an enlarged trocar site (Fig. 2).
3. Lower the pneumoperitoneum to 7 mmHg, observe for 3 minutes to confirm that there is no active bleeding, then remove the machine arm, pull out the Trocar, deflate, and suture the abdominal incision.
4. Postoperative observation: Postoperative analgesia was administered with intramuscular meloxicam (0.2 mg/kg once daily for 3 days). Pigs were left with gastric tube for 7 days and were fed with parenteral nutrition, and the veterinarians closely monitored and examined the pigs three times a day. Monitoring continued for 21 days postoperatively. Blood was drawn on the first day of postoperative period for routine blood test and biochemical indexes, which was repeated on the seventh day of postoperative period. Humane endpoints were predefined as: > 15% body-weight loss, sustained anorexia >24 h, severe respiratory distress, inability to ambulate, or signs of uncontrolled pain despite analgesia. Animals meeting humane endpoints would be euthanized immediately using intravenous pentobarbital sodium. Euthanasia was performed at the end of the experiment.

3. Methods of executing animals at the end of the experiment

Intravenous euthanasia agents.

### Outcomes

Efficacy evaluation: The primary efficacy index was the surgical success rate, which was defined as completing the robotic surgery as planned and not switching to another procedure. Secondary efficacy was intraoperative blood loss and the operative time that was decomposed into (1) positioning time: from trocar placement to robot docking; (2) robotic arm operation time: duration of active robotic manipulation; and (3) total surgery time: skin incision to skin closure.

Safety evaluation: Anesthesia time, heart rate, SpO_2_, complications (infection, hematoma, prolonged incision healing time, incision split, anastomotic obstruction, anastomotic fistula and digestive dysfunction), unnecessary injury to animals and systematic violation of procedures.

Feasibility evaluation: The surgeon’s satisfaction with the operation of the robotic system: (1) assessment of equipment docking task load: using the NASA-TLX measure-ment scale; and (2) intraoperative operating sensation score. Surgeon workload was evaluated using the standard NASA Task Load Index (NASA-TLX), which includes six dimensions: mental demand, physical demand, temporal demand, performance, effort, and frustration. A raw NASA-TLX approach was used. Each dimension was scored as originally proposed by Hart and Staveland, where higher scores indicate greater workload. The overall workload score for each procedure was calculated as the unweighted mean of the six dimension scores.

### Statistical analysis

Statistical analysis was performed using SPSS 25.0; GraphPad Prism (version 8, GraphPadSoftware, San Diego, USA). Repeated-measures ANOVA was used to analyze laboratory parameters across three time points (preoperative, postoperative day 1, postoperative day 7). Sphericity was evaluated using Mauchly’s test; when violated, the Greenhouse–Geisser correction was applied. Post-hoc pairwise comparisons were adjusted using Bonferroni correction to control for multiplicity. Also, the data were expressed as mean ± SD. All statistical analyses were performed using a two-tailed test with α = 0.05.

## Results

All eight pigs participating in this study were 3 months old; three males and five females; and in good health. Body weight was 41.63 ± 5.01 (35–50) kg (Table1). Partial gastrectomy was successfully completed in all animals and systematically evaluated for its efficacy, safety, and feasibility as follows:

1. Efficacy evaluation

**Table 1:** Basic information.

	1	2	3	4	5	6	7	8
Weight (kg)	37	45	42	43	37	50	35	44
Age (M)	3	3	3	3	3	3	3	3
Sex	Female	Female	Female	Female	Male	Female	Male	Male
Health	Excellent	Excellent	Excellent	Excellent	Excellent	Excellent	Excellent	Excellent

All surgeries were completed as planned, with no intermediate open or intraoperative complications, and the surgical success rate was 100% (Table 2). The average operation time was 76.75 ± 19.07 min (range: 50–105 min), of which the positioning time was 11.13 ± 3.14 min, and the operation time of the robotic arm was 23.00 ± 8.62 min. Intraoperative bleeding was 4.75 ± 0.46 ml, which was in line with the requirements of minimally invasive surgery. The survival rate of the animals after surgery was 100%, and no death or serious adverse events occurred during the perioperative period.

2. Safety evaluation

**Table 2:** Efficacy evaluation.

	1	2	3	4	5	6	7	8	
Surgical success rate	√	√	√	√	√	√	√	√	100%
Conversion to open surgery rate	-	-	-	-	-	-	-	-	0%
Perioperative mortality rate	-	-	-	-	-	-	-	-	0%
Total surgery time (min)	97	90	65	61	105	77	50	69	76.75 ± 19.07
Positioning time (min)	10	13	6	10	11	17	10	12	11.13 ± 3.14
Robotic arm operation time (min)	32	29	31	10	11	24	20	27	23.00 ± 8.62
Intraoperative blood loss (mL)	5	5	4	5	5	5	5	4	4.75 ± 0.46

The vital signs of the animals were stable during the operation, the heart rate averaged 75.38 ± 8.31 bpm, the blood oxygen saturation (SpO_2_) averaged 96.38 ± 1.60%, and the anesthesia time was 102.25 ± 24.94 min (Table 3). No infection, hematoma, prolonged incision healing time, incision split, anastomotic obstruction, anastomotic fistula, and digestive dysfunction occurred in all animals, and the anastomosis healed well (Table 3). No nonessential injuries were caused to the experimental animals during the operation, and no systematic irregularities occurred.

**Table 3:** Safety evaluation.

	1	2	3	4	5	6	7	8	
Anesthesia Time (min)	130	118	122	71	115	104	60	98	102.25 ± 24.94
Heart Rate (bpm)	88	85	68	71	68	68	73	82	75.38 ± 8.31
SpO_2_ (%)	99	98	96	96	96	94	97	95	96.38 ± 1.60
Complications									
Infection	-	-	-	-	-	-	-	-	0%
Hematoma	-	-	-	-	-	-	-	-	0%
Delayed Incision Healing	-	-	-	-	-	-	-	-	0%
Incision Dehiscence	-	-	-	-	-	-	-	-	0%
Anastomotic Obstruction	-	-	-	-	-	-	-	-	0%
Anastomotic Leakage	-	-	-	-	-	-	-	-	0%
Digestive Dysfunction	-	-	-	-	-	-	-	-	0%
Unnecessary Injury to Animals	-	-	-	-	-	-	-	-	0%
Systematic Violation of Procedures	-	-	-	-	-	-	-	-	0%

**Figure 3.**
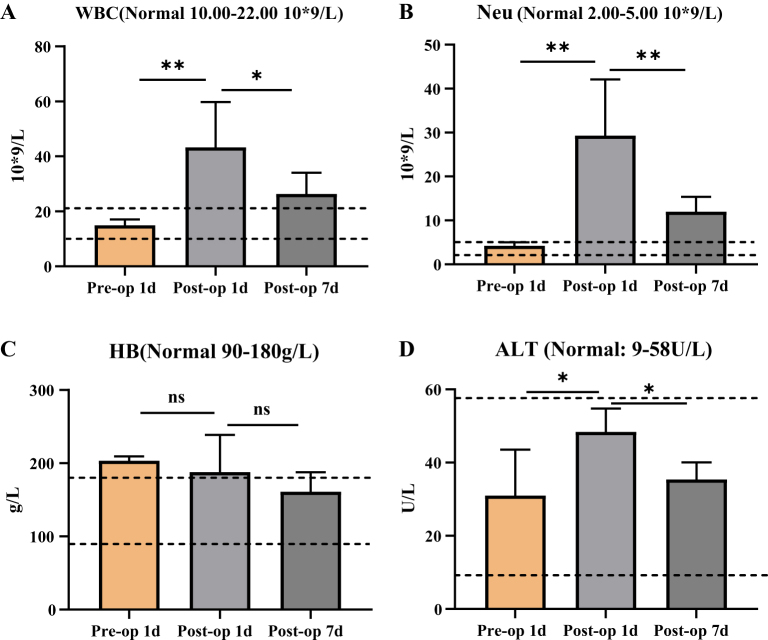
Perioperative laboratory parameter in a porcine model (*n* = 8; mean ± SD). (A) White blood cell (WBC). (B) Neutrophils (Neu). (C) Hemoglobin (HB). (D) Alanine transaminase (ALT).

The white blood cell (WBC) levels for preoperative Day 1, postoperative Day 1 and Day 7 were 14.95 ± 2.10 10*^9^/L, 43.32 ± 16.44 10*^9^/L and 26.33 ± 7.74 10*^9^/L, respectively (*P*_pre Day 1 vs post Day 1_ = 0.008, *P*_post Day 1 vs Day 7_ = 0.035). The Neu levels for preoperative Day 1, postoperative Day 1 and Day 7 were 4.23 ± 0.77 10*^9^/L, 29.30 ± 12.83 10*^9^/L and 11.99 ± 3.37 10*^9^/L, respectively (*P*_pre Day 1 vs post Day 1_ = 0.002, *P*_post Day 1 vs Day 7_ = 0.006). The HB levels for preoperative Day 1, postoperative Day 1 and Day 7 were 203.50 ± 5.90 g/L, 187.88 ± 50.83 g/L and 161.00 ± 26.71 g/L, respectively (*P*_pre Day 1 vs post Day 1_ = 0.851, *P*_post Day 1 vs Day 7_ = 0.446). The ALT levels for preoperative Day 1, postoperative Day 1 and Day 7 were 32.36 ± 3.38 U/L, 48.38 ± 6.41 U/L and 35.38 ± 4.69 U/L, respectively (*P*_pre Day 1 vs post Day 1_ = 0.018, *P*_post Day 1 vs Day 7_ = 0.021) (Fig. 3).

3. Feasibility evaluation

Operator task load was assessed by NASA-TLX scales and the results showed low overall load.The NASA-TLX scores of Mental Demand, Physical Demand, Temporal Demand, Performance, Effort and Frustration were 3.00 ± 1.31, 2.63 ± 0.52, 3.00 ± 1.07, 2.25 ± 0.71, 2.88 ± 0.84, and 2.25 ± 0.71 (Table 4). Intraoperative operation feeling scores were rated 50% as well and 50% as moderate.

**Table 4:** Feasibility evaluation.

	1	2	3	4	5	6	7	8	
NASA-TLX									
Mental Demand	1	2	3	2	4	5	4	3	3.00 ± 1.31
Physical Demand	2	3	3	2	3	2	3	3	2.63 ± 0.52
Temporal Demand	4	1	4	3	2	3	3	4	3.00 ± 1.07
Performance	2	2	3	1	3	2	2	3	2.25 ± 0.71
Effort	2	3	4	2	3	2	3	4	2.88 ± 0.84
Frustration	2	1	3	2	3	2	3	2	2.25 ± 0.71
Operation Feeling Score	well	well	moderate	well	moderate	well	moderate	moderate	

## Discussion

In this study, we verified the feasibility, effectiveness and safety of partial gastrectomy in animal experiments by completing gastric resection in a porcine model with the domestically produced SR2000 surgical robotic system. The results showed that all surgeries were completed as planned (100% success rate), with an average operative time of 76.75 minutes, intraoperative bleeding ≤5 ml, and a 100% postoperative survival rate, indicating that the system possesses high efficiency and stability in basic gastrointestinal surgery. In addition, its open console design significantly reduces the “physical demand” and “frustration” scores on the NASA-TLX scale by optimizing the operator’s neck posture and dual-screen navigation display, reflecting the advanced ergonomic design, which may help to Reducing operator fatigue and improving operational efficiency^[^17^]^.

WBC and Neu levels were transiently elevated on postoperative day 1, suggesting an acute inflammatory response, but significantly decreased to near normal levels on day 7. This trend is consistent with the pattern of postoperative inflammatory response in animals reported in the literature^[^18^]^, suggesting that the postoperative inflammatory response was manageable and did not affect vital organ function^[^19^]^. Liver function indices were not significantly abnormal pre- and postoperatively. In addition, the anastomosis healed well without complications such as infection and fistula, further validating the safety of the system, which is consistent with the advantages of robotic-assisted surgery in reducing tissue damage^[^20^]^.

This study not only demonstrates the technical feasibility, safety, and procedural stability of the Kangduo SR2000 in performing partial gastrectomy in a porcine model, but also provides a crucial translational bridge for its clinical adoption in gastrointestinal surgery^[^21^]^. The consistent completion of procedures without intraoperative conversion or major complications, coupled with minimal blood loss and stable perioperative physiological parameters, highlights the platform’s reliability in replicating complex laparoscopic maneuvers^[^22^]^. Importantly, the ergonomic advantages observed in the NASA-TLX assessment suggest that the SR2000 may help reduce surgeon fatigue and improve workflow efficiency^[^23^]^. These results are particularly significant in the context of advancing from the proven clinical track record of the first-generation SR1000 system toward broader indications in gastrointestinal oncology^[^24^]^.

By validating performance in a controlled animal model, this work lays the groundwork for multicenter clinical trials targeting a spectrum of gastric procedures, from early gastric cancer resections to total gastrectomy with lymphadenectomy. The findings also offer valuable feedback for iterative engineering improvements, such as enhancing haptic feedback and exploring single-port approaches, which would further expand its competitiveness in the global surgical robotics market^[^25,26^]^. Collectively, these contributions accelerate the trajectory of the Kangduo SR2000 from experimental validation to routine clinical application, supporting its role in driving the domestic surgical robotics industry from “following” to “leading” in the field of minimally invasive gastrointestinal surgery^[^27^]^.

The limitations of this study need to be interpreted with caution. First, the healthy porcine model could not mimic the pathological features of tumor patients (e.g., adhesions, vascular variations), and the anatomical differences between porcine and human digestive systems (e.g., mesenteric length, vascular distribution) may affect the clinical extrapolation of the surgical strategy. Secondly, the small sample size (*n* = 8) and short observation period (21 days) may have led to statistical bias to assess long-term complications (e.g., anastomotic stenosis). A formal power calculation was not performed because the study was designed as an exploratory feasibility assessment consistent with preclinical robotic validation studies. A sample size of eight animals is aligned with published feasibility frameworks for surgical robotics and was considered adequate to characterize operative workflow and perioperative safety. In addition, the type of surgery was limited to partial gastrectomy and did not cover complex procedures such as total gastrectomy or esophagojejunostomy^[^28^]^.

Future studies should expand the sample size, extend the follow-up time, and incorporate tumor models to evaluate the precision of the system in the execution of the tumor-free principle and lymph node dissection. On the technical level, the integration of 5G teleoperation and preoperative AI navigation (e.g., 3D tumor border reconstruction) can further enhance the level of intelligence^[^29,30^]^. At the same time, supporting simplified training systems (e.g., virtual reality simulators) and optimized equipment maintenance processes are needed to promote the widespread use of domestic robots in primary care.

In addition to the technical feasibility demonstrated in this study, the SR2000 system also offers notable economic advantages. As a domestically developed platform, its acquisition and maintenance costs are substantially lower than those of imported robotic systems such as the da Vinci Xi. This cost reduction may enable wider adoption in secondary- and county-level hospitals in China, particularly in regions where high-cost platforms are not feasible.

Furthermore, the four-arm architecture, improved instrument triangulation, and dual-console capability of the SR2000 provide scalability for more advanced procedures beyond partial gastrectomy, including D2 lymphadenectomy, esophagogastric reconstruction, Roux-en-Y gastric bypass, and hepatopancreatobiliary operations. These features position the SR2000 as a platform capable of expanding procedural repertoire as surgeon proficiency and training programs continue to mature.

To sum up, the Kangduo robot system has demonstrated the potential to replace imported equipment in animal experiments, but its technical maturity and clinical applicability still need to be verified through multi-center clinical trials. Through continuous optimization of human–machine interaction and expansion of functional modules, the domestic surgical robot is expected to achieve breakthrough progress from “following” to “leading” in the field of minimally invasive surgery.

## Data Availability

The data that support the findings of the present study are available from the corresponding author upon reasonable request.
